# Mainstreaming female genital schistosomiasis to ensure it is not neglected among the neglected tropical diseases

**DOI:** 10.1017/S0031182025100838

**Published:** 2025-12

**Authors:** Francisca Mutapi, Helmi Hietanen, Takafira Mduluza

**Affiliations:** 1 Institute of Immunology and Infection Research, University of Edinburgh, Ashworth Laboratories, Edinburgh, UK; 2 Tackling Infections to Benefit Africa (TIBA) Partnership, University of Edinburgh, Ashworth Laboratories, Edinburgh, UK; 3 Department of Biotechnology and Biochemistry, University of Zimbabwe, Harare, Zimbabwe

**Keywords:** diagnostic tools, environmental management, female genital schistosomiasis, health education, neglected tropical diseases, preventative chemotherapy, sanitation and hygiene, schistosomiasis, water, women and girls

## Abstract

Female genital schistosomiasis (FGS) is a neglected manifestation of *Schistosoma haematobium* infection, affecting an estimated 56 million women in sub-Saharan Africa. It is characterized by lesions in the genital tract, leading to symptoms like pain, infertility and an increased risk of HIV transmission. Despite its prevalence, FGS remains underdiagnosed and underreported due to limited awareness and diagnostic capabilities. Current knowledge emphasizes the need for integrated approaches combining diagnosis, treatment with praziquantel and education. There are ongoing efforts to integrate FGS services into women’s sexual and reproductive services, yet to date many African countries lack programmatic guidance to achieve this. More comprehensive integration and mainstreaming of FGS prevention, control and treatment across various sectors is needed to ensure intersectoral collaboration and financing of programmes. This review examines the various intervention tools currently available to achieve FGS integration in health systems. These include water, sanitation and hygiene improvements, environmental management, health education and inclusion of preschool-aged children in national schistosomiasis control programmes. Highlighted are also the required diagnostic and therapeutic tools, preventive interventions, effective policy and sustainable funding, all integral to achieving comprehensive FGS mainstreaming.

## Graphical abstract

**Figure:**
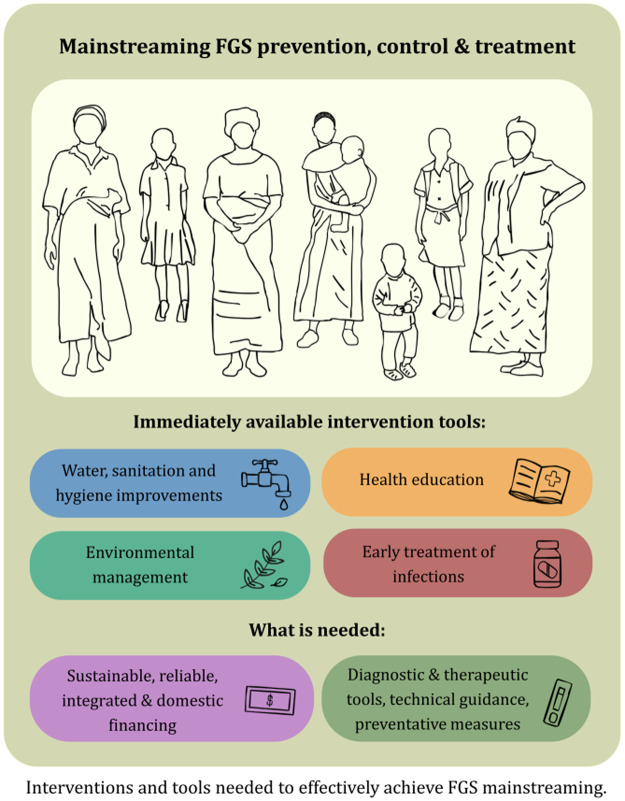

## Introduction

Mainstreaming the control and treatment of female genital schistosomiasis (FGS) is crucial due to its high prevalence, affecting an estimated 56 million women in sub-Saharan Africa (Mberu et al. 2025), and its severe impact on women’s health, causing genital lesions, pain, bleeding and infertility (Hotez et al. 2009). Additionally, FGS significantly increases the risk of HIV transmission by up to 3-fold due to mucosal barrier disruption (Kjetland et al. 2006). Integrating FGS into standard reproductive and sexual health services would not only ensure timely diagnosis and treatment but also reduce HIV transmission rates and promote gender equity by addressing a disease that disproportionately affects young and economically disadvantaged women (World Health Organization, 2015).

Recent meta-analyses have reinforced the association between *Schistosoma haematobium* infection and FGS with Patel et al. (2021) confirming a strong link between urogenital schistosomiasis and gynaecological manifestations such as contact bleeding, vaginal discharge and lesions, while Zirimenya et al. (2020) demonstrated that FGS significantly increases the risk of HIV acquisition among affected women.

Low awareness and frequent misdiagnosis of FGS as sexually transmitted infections (STIs) highlight the need for its inclusion in reproductive health education and healthcare worker training to improve accurate diagnosis and reduce stigma (Secor, 2012). Additionally, treating FGS with praziquantel (PZQ) is highly cost-effective, making its integration into existing health services, such as deworming and HIV care, an efficient use of resources (Fenwick et al. 2009). Effective control of FGS would also support Sustainable Development Goals (SDGs), particularly SDG 3 (Good Health and Well-being) and SDG 5 (Gender Equality), by addressing a neglected tropical disease (NTD) that has long been overlooked. Mainstreaming FGS control into national health systems is essential for providing equitable, sustainable and efficient healthcare to millions of women in endemic regions. There are current efforts to integrate FGS services into women’s sexual and reproductive services (Global Schistosomiasis Alliance, 2025). Nonetheless, this has been limited due to a combination of low awareness, inadequate funding, diagnostic challenges, fragmented health systems, stigma, insufficient healthcare worker training and weak policy frameworks. Awareness among healthcare providers and policymakers is low, leading to frequent misdiagnosis as STIs (Hotez et al. 2009). Funding for FGS is heavily reliant on external donors, with limited domestic resources, and recent cuts in UK and USAID funding have further strained control programmes (World Health Organization, 2021). Diagnostic tools for FGS are often inaccessible or too costly, complicating accurate diagnosis and integration into reproductive health services (World Health Organization, 2015). Health systems in many countries remain fragmented, preventing effective integration of FGS treatment into existing sexual and reproductive health (SRH) programmes (World Health Organization, 2021). Additionally, socio-cultural stigma surrounding genital symptoms deters women from seeking treatment, while limited training for healthcare workers exacerbates misdiagnosis and missed treatment opportunities (Wei et al. 2012). The absence of specific national policies or guidelines for FGS control further complicates efforts to secure resources and coordinate responses effectively (World Health Organization, 2021).

This review identifies health system entry points for mainstreaming FGS prevention, control and treatment, and discusses current challenges and opportunities.

## Male genital schistosomiasis

Although the focus of this review is FGS, it is important to note that urogenital schistosomiasis also affects men and it is worth including summary details of the condition here. Male genital schistosomiasis (MGS) is a similarly under-recognized manifestation of *S. haematobium* infection, with growing evidence of its clinical and public health significance in endemic African settings. MGS affects various components of the male genital tract, including the prostate, seminal vesicles and epididymis, and has been associated with haemospermia, pelvic discomfort, infertility and increased risk of HIV transmission (Leutscher et al. 2008; Kayuni et al. 2018). Studies among fishermen in Malawi have reported high prevalence of MGS-related symptoms, along with poor awareness and limited access to appropriate diagnosis and treatment (Kayuni et al. 2021). Notably, a recent prospective study found that MGS may influence seminal HIV-1 shedding even in men on antiretroviral therapy, suggesting important implications for HIV prevention strategies (Kayuni et al. 2023). Despite these findings, MGS remains largely neglected in research, policy and control programmes, underscoring the urgent need for integrated diagnostic, treatment and health education interventions (Kayuni et al. 2018). Screening studies among African migrants in non-endemic settings further reveal long-term complications and chronic morbidity linked to undiagnosed MGS, including erectile dysfunction and infertility (Roure et al. 2024). Addressing MGS through systematic inclusion in NTD control frameworks could also yield significant reproductive and sexual health benefits for affected populations.

## Immediately available FGS intervention tools

### FGS prevention, control and management

While general control strategies for *S. haematobium* such as mass drug administration (MDA) and water, sanitation and hygiene (WASH) interventions are essential, the prevention and management of FGS require additional targeted approaches. These include integrating FGS education into community outreach and SRH services to reduce stigma and increase early care-seeking (Umbelino-Walker et al. 2023). Diagnosis remains challenging, as standard urine-based tests often fail to detect genital involvement; thus, visual inspection via colposcopy or digital imaging is recommended in endemic settings (HPV Global Action, 2022; World Health Organization, 2022a). PZQ remains the treatment of choice, with evidence supporting both therapeutic and preventive administration in girls and women at risk – even in the absence of confirmed urinary schistosomiasis (World Health Organization, 2022b; Preston et al. 2023). The Female Schistosomiasis and sexual and reproductive health and rights (SRHR) Training (FAST) package exemplifies an integrated care model, combining provider training, visual diagnosis and treatment delivery within existing HIV, cervical cancer and maternal health platforms (Krentel et al. 2024; FAST Package, 2025). Strengthening national policies and coordinating multisectoral responses through initiatives such as the FGS Integration Group are also vital to mainstream FGS into health systems (Global Schistosomiasis Alliance, 2025). Together, these strategies highlight the need for a gender-sensitive, integrated approach to FGS control.

Nonetheless, in order to mainstream FGS, it is essential that FGS prevention, control, treatment and management are integrated into the currently running programmes for schistosomiasis control as well as SRHR as detailed below.

### Improving water, sanitation and hygiene

To prevent children, girls and women from getting infected with schistosomes, several key actions are necessary. Improvements in WASH are essential, as contaminated water sources are the primary transmission route for schistosomiasis in Africa. From studies in Malawi, it is already known that improvements in water access and sanitation contribute to significant reductions in schistosome infection levels and transmission, particularly in areas with high reliance on untreated water sources for daily activities (Grimes et al. 2015) and yet WASH coverage remains poor in African countries. As of 2022, only 31% of the African population had access to safely managed sanitation services (World Health Organization, 2023b). For safe drinking water, approximately 39% of the population used safely managed services as of 2020 (United Nations Children’s Fund, 2022). The urban-rural divide is stark: in urban areas, about 66% of people lack safely managed sanitation, while in rural areas this figure rises to 75% (World Health Organization, 2023b). For drinking water, the gap is even more pronounced, with 40% of urban residents lacking safely managed drinking water compared to 75% in rural areas (United Nations Children’s Fund, 2022). These statistics highlight the persistent challenges in providing adequate WASH across Africa. Without addressing these, millions of women and girls who are often disproportionately exposed to infection remain at risk of FGS. WASH should be mainstreamed into infrastructure and national development plans, local government policies and community-based strategies. This can be achieved by integrating WASH-related goals into broader health, education and rural development programmes, ensuring that funding is allocated and resources are consistently mobilized. For example, countries like Ethiopia and Kenya have incorporated WASH objectives into their national poverty reduction and public health strategies (World Health Organization, 2021).

### Environmental management

Beyond WASH, there is a need for environmental management programmes to address occupational risk of infection (Secor, 2012). Agricultural work often involves prolonged contact with contaminated freshwater sources, putting people in endemic countries at risk of contracting urogenital schistosomiasis. This occupational exposure has been documented in several African countries (Ayabina et al. 2021).

Commercial irrigation schemes can expose workers to *S. haematobium* infection. These schemes can be improved to prevent schistosomiasis transmission through an integrated approach that combines engineering, agricultural and health interventions. A successful example of this is the Small Holder Irrigation Project implemented in Zimbabwe in the late 1980s and early 1990s (Chimbari, 2012). This can be achieved by mainstreaming disease prevention as part of agriculture, civic and water management programmes. The Mushandike Programme is an example of this intersectoral collaboration. In this case, toilets were constructed in such a way that workers were closer to a toilet than to a bush at all times; alternate water storage in canals meant segments were left dry to avoid snail colonization and/or proliferation (Chimbari, 2012).

### Health education

Health education is critical to empower people exposed to schistosomiasis and at risk of FGS to make informed choices and any behavioural changes. For example, health education programmes targeting communities, especially children and women, are vital in promoting behaviours such as avoiding swimming in contaminated water, using sanitation facilities and practising environmentally secure hygiene (Hotez et al. 2009; Stothard et al. 2025). These can easily be integrated in schools, for example, Tanzania has implemented a health education project focused on personal hygiene and the control of schistosomiasis and helminth infections in primary schools (Lansdown et al. 2002). There is a need to extend these programmes to community areas to target out-of-school people at risk.

The education and WASH facilities need to be context-specific. It has been previously shown that WASH uptake and compliance is not simply a matter of having access to toilets and safe water: gender-related socio-cultural reasons also determine whether or not communities use toilets (Lampard-Scotford et al. 2022). It is also known that various factors including education and awareness influence health-seeking behaviour in rural Africa (Okojie and Lane, 2020), and these may influence when and where a woman seeks health inventions for FGS. Thus, for women and girls in particular, gender-sensitive approaches that address specific barriers to access and care are necessary, ensuring that they have equal opportunities to benefit from prevention and treatment programmes (World Health Organization, 2015).

### Diagnosing FGS

Current diagnostic approaches for FGS in Africa increasingly emphasize feasibility, accessibility and integration into existing health services. While traditional diagnosis relies on colposcopic identification of characteristic lesions (e.g. sandy patches and rubbery papules), this method requires specialist equipment and training, which are often unavailable in endemic areas (Jacobson et al. 2022). To address this, simplified visual inspection methods, including the use of hand-held digital cameras and mobile colposcopy tools, have been introduced and adapted for use by trained mid-level health workers (Rafferty et al. 2021; Lamberti et al. 2024a). Tools such as the World Health Organization’s (WHO) FGS pocket atlas (World Health Organization, 2015) and the training modules in the FGS-SRH FAST package have enabled nurses and community health workers to recognize and respond to FGS in primary care settings (Gyapong et al. 2024; Krentel et al. 2024). Symptom and risk-factor checklists are also being piloted as community-based screening tools, allowing affected individuals to report key symptoms – such as genital itching, abnormal discharge or dyspareunia – to healthcare professionals, although specificity remains limited (Mbwanji et al. 2024).

### Early treatment of infections

Late-stage FGS complications are currently not treatable by PZQ. It is therefore best to treat women and girls early in order to ensure infection clearance and reversal of early pathology. A recent clinical trial in Madagascar showed that PZQ treatment had limited effectiveness in treating established FGS-associated cervical lesions (Arenholt et al. 2024). While the treatment showed some efficacy in reducing abnormalities observed during a pelvic exam, the urogenital complaints, parasite biomarkers and cervical lesions were refractory to PZQ treatment. This is not surprising as the drug primarily targets the parasites, although it has been shown by us and other researchers that the removal of the worms itself modulates the host immune responses (Mutapi et al. 1998; Chaponda and Lam, 2024). Considering the costs associated with managing late-stage FGS and its impact on physical and mental health, the relative cost of early treatment is cheaper, currently costing between $0·6 and $4·46 per person treated (Salari et al. 2020).

Preventative chemotherapy (PC), that is, treatment of at-risk populations in endemic settings at regular intervals (World Health Organization, 2025) is currently the most widely used tool for controlling schistosomiasis infections. It has been effective in reducing infection levels in Africa over the past two decades. For example, infection prevalence has decreased by almost 60% across sub-Saharan Africa, primarily due to PZQ PC (Kokaliaris et al. 2022). This reduction in prevalence has been accompanied by a decrease in infection intensity, particularly in school-aged children (World Health Organization, 2023a).

### Preventative chemotherapy challenges

**Figure 1.**
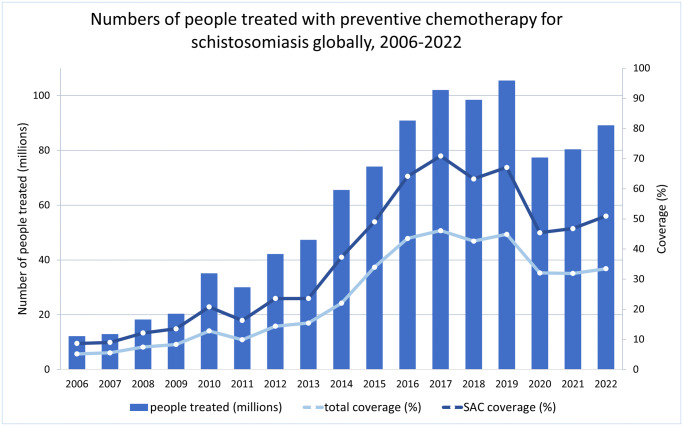
Numbers of people treated with preventative chemotherapy for schistosomiasis globally between 2006 and 2022. Adapted from the World Health Organization (World Health Organization, 2023c). SAC, school-aged children.

While these advances have been welcome, there are continuing challenges due to insufficient PC coverage. In 2018, approximately 76·2 million school-aged children and 19·1 million adults were still treated with PZQ in Africa, indicating a continued need for treatment (Kokaliaris et al. 2022). Figure 1 below shows the variable coverage of schistosomiasis PC.

**Figure 2.**
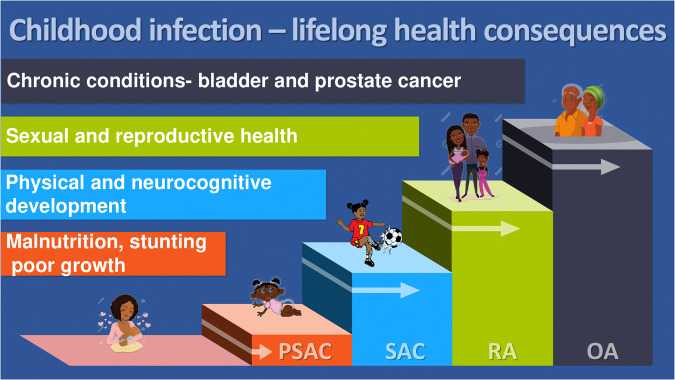
Progression of the consequences of untreated schistosome infection with age. PSAC, preschool-aged children (≤5 years old); SAC, school-aged children (6–15 years); RA, adults of reproductive age (16–50 years old); OA, older adults (51+).

Second, despite current WHO guidelines (Lo et al. 2022) recommending inclusion of all age groups (preschool children, schoolchildren and adults) in national schistosomiasis control programmes, these programmes are focusing on school-aged children, typically 6–15 years, and some adults through community-based MDA. This means an estimated 50 million preschool-aged children (PSAC) (<6 years) continue to be excluded from national schistosomiasis control programmes (Mutapi et al. 2024). Apart from perpetuating a health inequality, this is a lost window of opportunity to treat early infections before they progress to FGS complications and other chronic pathologies as shown in Figure 2. A cross-sectional study conducted in Angola among 245 PSAC found a prevalence of urogenital schistosomiasis of 30·2% in this age group (Sánchez-Marqués et al. 2023).

To address this, there is a need to include PSAC treatment in national schistosomiasis control programmes. This will be more effectively done by mainstreaming PSAC treatment into health system components serving child health and/or development. The recent development and WHO prequalification of a paediatric formulation of PZQ, arpraziquantel (arPZQ), should accelerate the inclusion of PSAC into national programmes.

To date, PSAC in Uganda have been the first to receive arPZQ through implementation studies under the ADOPT program, with preparatory rollout activities in Kenya, Côte d’Ivoire, Tanzania, Senegal and Zimbabwe (Pediatric Praziquantel Consortium, 2025a, 2025b; The Special Programme for Research and Training in Tropical Diseases (TDR), 2025) underway to enable broader scale-up. However, while the rest of Africa await the adoption of this new formulation, operational studies are underway on optimizing the use of the currently available adult tablets; for example, a recent study in Uganda showed that 80 mg/kg body weight given as two 40 mg/kg doses three hours apart was safe and more effective in clearing infection than the current single dose of 40 mg/kg alone in this age group (Bustinduy et al. 2025).

Whilst solutions to some of the challenges in FGS control are pending, we have detailed immediately actionable priorities in Table 1. These can be implemented in NTD programmes and community health services to improve integration, health worker training, community awareness and education, and access to diagnosis and treatment.

**Table 1.** Top 5 actionable priorities for FGS control for NTD programme managers and community health workers

Priority	Why it’s a priority	Implementation approach
1. Integration of FGS screening into routine SRH and primary healthcare services.	FGS symptoms overlap with STIs and other conditions, often leading to misdiagnosis. Integration ensures early detection and treatment.	Incorporate FGS symptom checklists (e.g. WHO’s Pocket Atlas) into SRH, HIV and maternal health clinic protocols.
2. Training health workers to recognize and manage FGS.	Lack of awareness among health workers leads to under-diagnosis and mistreatment.	Roll out simple, pictorial training materials for community health workers (CHWs) and nurses, including symptom identification and referral pathways.
3. Community awareness campaigns and school-based education.	Women and girls often do not understand the disease or its waterborne transmission.	Use edutainment including local radio, schools and community meetings to deliver culturally sensitive FGS prevention and awareness messages.
4. Improved access to praziquantel, especially for PSAC, adolescent girls and women.	PSAC, women and older girls out of school are often excluded from school-based deworming programmes.	Distribute praziquantel via CHWs, antenatal clinics and routine SRH service points.
5. Development and use of syndromic management and point-of-care tools.	Many areas lack lab capacity; symptoms are non-specific, and diagnosis is delayed.	Adopt low-cost checklists and invest in validated point-of-care diagnostic tools usable by CHWs.

## Going forward: tools needed

A recent series of consultative workshops conducted in Zimbabwe and Tanzania involved women affected by FGS, the local primary health workers and gynaecologists serving them, and health policymakers (Tackling Infections to Benefit Africa (TIBA) Partnership, 2024). The participants highlighted several current tool gaps, and made a call for action to researchers and funders to close these gaps to facilitate the prevention, treatment and management of FGS.

Currently, there is no universally accepted diagnostic reference standard for FGS. Methods, such as visual inspection via colposcopy, urine microscopy and molecular tests have limitations in accuracy, feasibility and accessibility, especially in endemic rural areas (Søfteland et al. 2024). Microscopy of cervicovaginal lavage sediments has recently been explored for schistosome egg detection in FGS patients, yet this also insufficiently distinguishes FGS cases (Stothard et al. 2025). Visual diagnosis using colposcopy is widely considered a reference standard but as highlighted earlier, it is often unavailable in resource-limited settings. Moreover, there is low inter-observer agreement among experts reviewing colposcopic images, highlighting the need for more reliable diagnostic methods (Sturt et al. 2023). Emerging artificial intelligence-based diagnostics – including smartphone-integrated image recognition tools and low-cost automated microscopes – are under exploration to address these limitations in FGS diagnostics, with studies demonstrating high accuracy in detecting *S. haematobium* eggs via digital imaging and deep learning models (Rubio Maturana et al. 2024). Ultimately, integrated training, visual aids and syndromic screening offer practical, scalable approaches for FGS detection in endemic African regions, while research continues to refine point-of-care (POC) diagnostic tools.

The WHO has emphasized the development of simple, affordable diagnostic tools for FGS as part of its 2021–2030 roadmap for eliminating NTDs (World Health Organization, 2021). Integrating FGS diagnostics into reproductive health services has been identified as a critical step towards addressing this neglected disease. The target product profile for FGS diagnostics should include the need for rapid POC diagnostics for all stages of FGS (World Health Organization, 2021).

The need to link diagnostics to treatment is critical. Participants at the two workshops indicated that the lack of treatment following diagnosis was a deterrent for seeking healthcare. Currently, there is no treatment for late-stage FGS symptoms. Advanced FGS often requires variable multidisciplinary management, including invasive procedures, to deal with morbidities and complications (Rossi et al. 2024). There is a clear need for research to determine optimal clinical treatment for FGS, potentially exploring higher and more prolonged doses of PZQ, anti-inflammatory drugs and possibly new combination therapies (Engels et al. 2020).

In order to deploy treatments, clear technical guidance for effective FGS care pathways implementable within local health systems is needed. The COUNTDOWN project has created and validated screening tools for FGS, including environmental risk assessment, discharge charts and symptom evaluation (COUNTDOWN, 2025). These need to be extended and adapted into locally relevant care pathways with accompanying health policies developed. It is noteworthy that to date there is no programmatic guidance for the integration of FGS and SRHR interventions in the way of a Minimum Service Package in African countries (Pillay et al. 2024).

There is also a need of strengthening preventative approaches through the development of drugs targeting the infective stage of the parasites (El Ridi and Tallima, 2013). Furthermore, there is need to develop tools that make water contacts safe for populations living in schistosome-endemic areas beyond the unrealistic wholescale mollusciciding of all infective water bodies. This includes mainstreaming anti-infection interventions such as cercaricidal soaps (Zhang et al. 2024) or topical lotions (e.g. dimethicone barrier cream) (Ingram et al. 2002) as well as anti-infective vaccines. While there are no current vaccines against human schistosomiasis, there are already candidates for cercaricidal soaps and creams. There is need to understand why these have not been widely adopted as public health measures. Undoubtably, cost will be a contributing barrier, hence the need for purposeful domestic financing.

## Financing female genital schistosomiasis control

There is currently a significant gap in the treatment coverage for schistosomiasis, with about two-thirds of the African population requiring schistosomiasis treatment not receiving it in 2022 (Garba, 2023). This is largely due to a funding gap for MDA. Challenges faced by African countries in financing schistosomiasis control include fragmented health financing, insufficient domestic budgets and competing priorities. Many countries rely heavily on external funding from organizations like the WHO and formerly USAID for MDA and awareness campaigns. In contrast, few have allocated domestic resources for schistosomiasis control, let alone specifically for FGS management. The calls for integrated FGS funding (FGS Integration Group, 2024) will only have impact on the people affected if sustainable and reliable funding is also identified. Ultimately, this requires strengthening health systems financing through increased domestic funding, integrated care models and leveraging public-private partnerships. These are essential for sustainable FGS control. Some African countries like Tanzania (George et al. 2023) have started allocating domestic funds for NTD control programmes, but more needs to be done at regional and continental levels.

For scalability, a health economic model – the SCREEN framework – has already been published, providing a structured approach to assess the cost-effectiveness and implementation feasibility of FGS interventions in low-resource settings (Lamberti et al. 2024b).

The implementation of initiatives such as the African Union Development Agency - New Partnership for Africa's Development (AUDA-NEPAD) ‘Prioritizing SRH to accelerate progress towards Universal Health Coverage’ (AUDA-NEPAD, 2025) and the financing through the domestic health financing efforts (AUDA-NEPAD, 2022) will progress FGS control and management.

## Conclusions

Various studies in Africa have clearly demonstrated that FGS is an important disease significantly affecting both the physical and mental health of millions of African women and girls (Masong et al. 2024). The lack of affordable sensitive and specific rapid POC diagnostics and therapeutics for advanced FGS is compromising health provision. Researchers and funders need to respond to calls both from people living with the disease (affected voices) and the WHO to develop not only these tools, but also interventions that make contact with infective water safe. In terms of improving awareness and care for people at risk of FGS or already suffering the clinical symptoms, there is urgent need for mainstreaming FGS programmes not only in SRH services but across the entire health system. Integrating disease prevention and control into broader health, education and rural development programmes such as agriculture, water and environmental programmes will ensure multisectoral engagement and a broader financing base.

Work in Zambia has already demonstrated a successful model integrating FGS management in a local health centre as well as providing community outreach and FGS awareness activities (Sturt et al. 2020; London School of Hygiene and Tropical Medicine, 2025). Furthermore, ongoing research efforts include a large longitudinal study in Zambia on FGS, human papillomavirus and other STIs, exemplifying how high-specificity diagnostics can be leveraged to address overlapping disease burdens (Shanaube et al. 2024). The third pillar of the WHO NTD Roadmap 2021–2030 focuses on changing operating models and culture to facilitate country ownership of NTD programmes. This is critical to deliver sustainable FGS prevention, control and eventual elimination as a public health problem. International funding, advocacy and leadership on FGS have made significant contributions to reducing the disease burden, including, for example, the initiative funded by the German government to support the integration of FGS diagnosis and treatment into SRH services (Global Schistosomiasis Alliance, 2024) which was launched during the 2024 World Health Summit. However, ultimately African countries need to take the lead and ownership of FGS programmes along with the rest of the NTDs to guarantee sustained health provision to their populations (Speak Up Africa, 2024).
